# Characteristics of Cr-B Coatings Produced on Vanadis^®^ 6 Tool Steel Using Laser Processing

**DOI:** 10.3390/ma14102621

**Published:** 2021-05-17

**Authors:** Aneta Bartkowska

**Affiliations:** Faculty of Materials Engineering and Technical Physics, Institute of Material Science and Engineering, Jana Pawla II 24, 60-138 Poznan, Poland; aneta.bartkowska@put.poznan.pl; Tel.: +48-616-653-572

**Keywords:** chromium coating, boron coating, Cr-B coating, laser alloying, microhardness, corrosion resistance

## Abstract

The paper presents the results of a study of the microstructure, chemical composition, microhardness and corrosion resistance of Cr-B coatings produced on Vanadis 6 tool steel. In this study, chromium and boron were added to the steel surface using a laser alloying process. The main purpose of the study was to determine the impact of those chemical elements on surface properties. Chromium and boron as well as their mixtures were prepared in various proportions and then were applied on steel substrate in the form of precoat of 100 µm thickness. Depending on the type of precoat used and laser processing parameters, changes in microstructure and properties were observed. Coatings produced using precoat containing chromium and boron mixture were characterized by high microhardness (900 HV0.05–1300 HV0.005) while maintaining good corrosion resistance. It was also found that too low laser beam power contributed to the formation of cracks and porosity.

## 1. Introduction

The laser is a universal high-energy source that can be used, among other purposes, for marking, cutting and modifying ferrous and nonferrous alloys, which is particularly interesting and discussed in many scientific papers [1,2,3,4,5]. Parts of machines and devices used in industry often have to meet stringent conditions. They result, among other factors, from high operating temperature, variable and cyclical nature of loads or aggressive and corrosive environment. These factors often create the necessity to use more and more advanced materials rich in alloying elements, which naturally leads to their price increase. This clearly reduces attractiveness of products and services offered. However, this disadvantage can be significantly reduced by using methods and techniques of surface engineering [6,7,8,9]. This field of technical sciences allows the use of inexpensive materials and refinement of their surfaces properties. This ensures high operational properties while maintaining relatively low product costs.

One of the most interesting methods allowing interference with surface layer properties is diffusion boriding. Such layers are characterized by high microhardness and good wear and corrosion resistance. However, the technique of creating a layer enriched with boron is quite time-consuming. The resulting layer, despite its good properties, can show cracking in the subsurface zone. Many scientists attempt to counteract this unfavorable phenomenon by introducing additional elements such as chromium or copper [10,11,12,13,14,15] in order to reduce brittleness. Laser modification is also used for this purpose [16,17,18]. One of the currently popular techniques is laser heat treatment [6,8,9], where the base material is alloyed with metals or nonmetals, including carbon [19], boron [20,21,22], chromium [23], silicon [23,24,25], molybdenum [26], carbides [27,28], borides [27] or mixtures of various elements [25,26,29,30,31,32,33,34,35,36]. As a result of the laser alloying process, the chemical composition and microstructure are changed, hence changing the properties of the processed material. This method is very attractive as it enables the control of many laser parameters, such as power, size of the laser beam diameter or scanning speed of laser beam. This allows for obtaining very different properties of the surface layer, adapted to the working conditions of specific machine parts.

In [20], the author stated that the microstructure and hardness of laser boronized tracks depend on laser treatment. The author stated that the microstructure formed in the surface layers consists of a solid solution, a boride eutectic and primary borides in various combinations, whereas the wear resistance of boronized steels increases with the growth in the amount of the eutectic or borides in the microstructure. Moriomto [21] analyzed influence of laser boronizing on microstructure and selected properties. The author observed that the boronized layers produced using 60 mass% FeB and 40 mass% B2O3 composite powder showed high microhardness values from 1134 to 1220 HV. In [34], the authors modified a surface of steel AISI 4135 H with laser remelting and injection of hard particles (tungsten or boron carbides) or with laser alloying using coatings of boron carbide or boron. The authors stated that at a slow feed rate a cellular microstructure of the remelted zone with dissolved carbides was created with a hardness of 750–820 HV. With larger powder feed rates, the remelted zone incorporated more carbides, but they were less dissolved and there was more porosity, and the resulting hardness was only 600 HV. When the feed rate is 9 g/min, the surface layers become brittle due to the presence of very hard, undissolved carbides. They state that the surface has a rippled morphology, which is too rough for industrial applications and may require machining after laser treatment. To improve laser track homogeneity, the alloying method was changed. Additionally, in order to improve surface finish, other elements with a higher density had been added to the boron paste. Zirconia was used in place of iron, which is heavier than boron but lighter than iron, and it can slow down the convection flow with a better distribution into the pool. When carbon borides were replaced with boron powder, the cracking phenomenon disappeared due to the reduction in carbon content. In [28], a laser surface alloying treatment on stainless steel using chromium carbide to improve wear resistance, oxidation and corrosion was presented. The isothermal oxidation test at 960 °C for 100 h showed that the surface alloying treatment improved oxidation resistance by about 100 times as a result of distribution of chromium carbides and increased chromium concentration. Dispersion and precipitation of chromium carbides in the remelted zone contributed to obtaining microhardness of about 1100 HV. Additionally, this type of surface alloying treatment of stainless steel improved wear resistance.

In [32], the microstructure of surface layer obtained by laser alloying of C45 steel with chromium and boron carbide was investigated. The surface of specimens was covered with mixture of chromium and boron carbide with the following component ratios in the coating: 1:3, 1:1 and 3:1, with a 0.2–0.3 mm thickness. Next, the specimens were treated with pulsed laser beam. When chromium was added to the boronizing coating the distribution of microhardness over the thickness of the fusion zone was uniform. However, the microhardness depends substantially on the amount of chromium in the coating. When the chromium content in the coating increases, the amount of borides forming in the process of laser treatment decreases, and in consequence hardness decreases. The authors showed that when chromium is contained in the boron-containing coating, the fusion zone has a ledeburite-like dendritic microstructure. They noticed that the microstructure of the surface layer obtained by laser borochromizing has high thermal stability, which means that after holding at 860 °C and subsequent cooling in air there is no substantial decrease in layer hardness.

A significant number of papers concern surface modification with only boron [16,17,18,21,22,26,35] or boron compounds such as oxides [21] or borides [27]. Therefore, it seems justified to raise the issue of modification by other chemical elements.

In this study, laser alloying of surface layer of Vanadis 6 steel with chromium and boron was investigated. The effects of these elements separately and together using various laser parameters were analyzed. The microstructure, microhardness, corrosion resistance and chemical composition of newly formed Cr-B coatings were investigated and compared.

## 2. Materials and Methods

**Figure 1:**
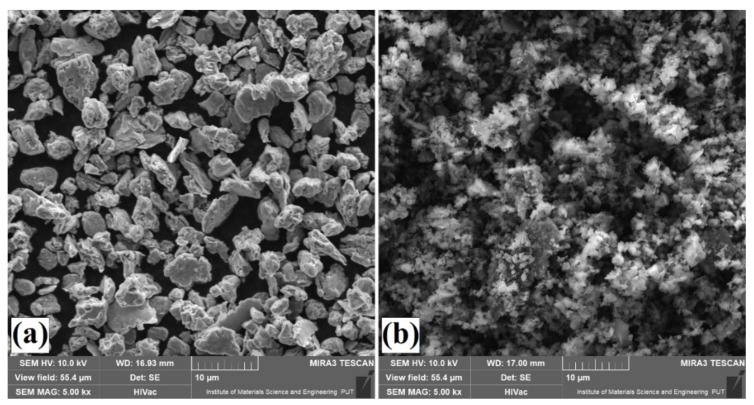
Morphology of chromium (**a**) and boron (**b**) powders.

Specimens made of Vanadis 6 tool steel were used. The chemical composition of this material, which was in accordance with the manufacturer’s data, is presented in Table 1. The morphology of chromium powder is presented in Figure 1a; its purity was 99.2%. The morphology of amorphous boron powder is shown in Figure 1b; its purity was ≥95%. The presented parameters are in accordance with the producer data (Sigma-Aldrich, Saint Louis, MO, USA).

Before laser alloying, the precoats in the form of paste were applied to the steel substrate. The composition proportions of the prepared precoats are summarized in Table 2. There were composed of powder or mixture of powders as well as adhesive material in the form of sodium water glass and distilled water. The thickness of precoat was 100 µm. The amount of powder was selected in a weight ratio while the water glass and distilled water were adjusted so that the applied precoat had adequate adhesion to the steel substrate. After drying the coating, the next step was laser processing.

Laser processing was carried out using Trumpf TruDiode 3006 diode laser with nominal power of 3 kW (TRUMPF, Ditzingen, Germany). The laser device worked with KR16-2 robotic arm (KUKA, Augsburg, Germany). In these studies, a variable laser beam power was used (550 W, 750 W, 950 W), but laser beam scanning speed of 50 mm/s was used throughout. Laser beam diameter was constant and was 1 mm. Laser tracks filled the entire surface of specimens, and the distance between the tracks was f = 0.4 mm; hence, overlapping was 60%. All processing parameters are presented in Table 3. For each laser beam power and for each precoat, 7 specimens were produced.

**Figure 2:**
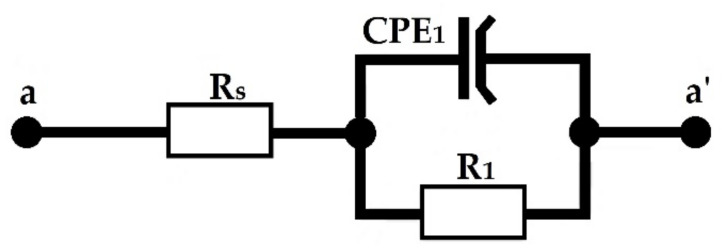
Electrochemical equivalent circuit: *R*_s_—solution resistance between the specimen and the reference electrode, *R*_1_—charge transfer resistance, CPE_1_—double layer capacitance.

Microstructure observations were carried out on polished and etched cross-sections of specimens with MIRA3 scanning electron microscope (TESCAN, Brno, Czech Republic). In the first step, the specimens were ground, then polished in diamond suspension and finally polished with a suspension of aluminum oxide. In order to conduct microstructure observations, the specimens were etched in 5% HNO_3_ solution. Chemical compositions of the coatings produced were investigated using an Ultim Max energy dispersive spectrometry (EDS) system (Oxford Instruments, High Wycombe, UK). To determine microhardness profiles of manufactured coatings, an FM-810 Vickers microhardness tester was used (Future-Tech, Kawasaki, Japan). The parameters of measurements were as follows: indentation load of 50 g (about 0.49 N) and loading time 15 s. Corrosion resistance of Cr-B coatings produced using laser alloying was determined in a 5% solution of NaCl at a constant temperature of 295 K where the rate of potential change was equal to 0.5 mV/s. The studies were performed on an ATLAS 0531 EU & IA potentiostat (Atlas-Sollich, Rębiechowo, Poland), which was equipped with AtlasCorr and AtlasLab software (version 2.24). Specimen polarization was carried out in the anodic direction in the potentials range from −1.5 to 1.5 V. In this study, the auxiliary electrode was made of platinum, while the reference electrode was a commercial saturated calomel electrode (SCE). Based on the analysis of current curves, the potentiodynamic corrosion and corrosion potential were determined. Corrosion test of the specimens was conducted in accordance with the PN-EN ISO 17475 standard. The electrochemical impedance spectroscopy (EIS) tests were performed with a 10 mV amplitude perturbation, and within the frequency range from 10^−3^ to 10^5^ Hz. The electrochemical equivalent circuit was used (Figure 2).

## 3. Results and Discussion

### 3.1. Microstructure and Chemical Composition

**Figure 3:**
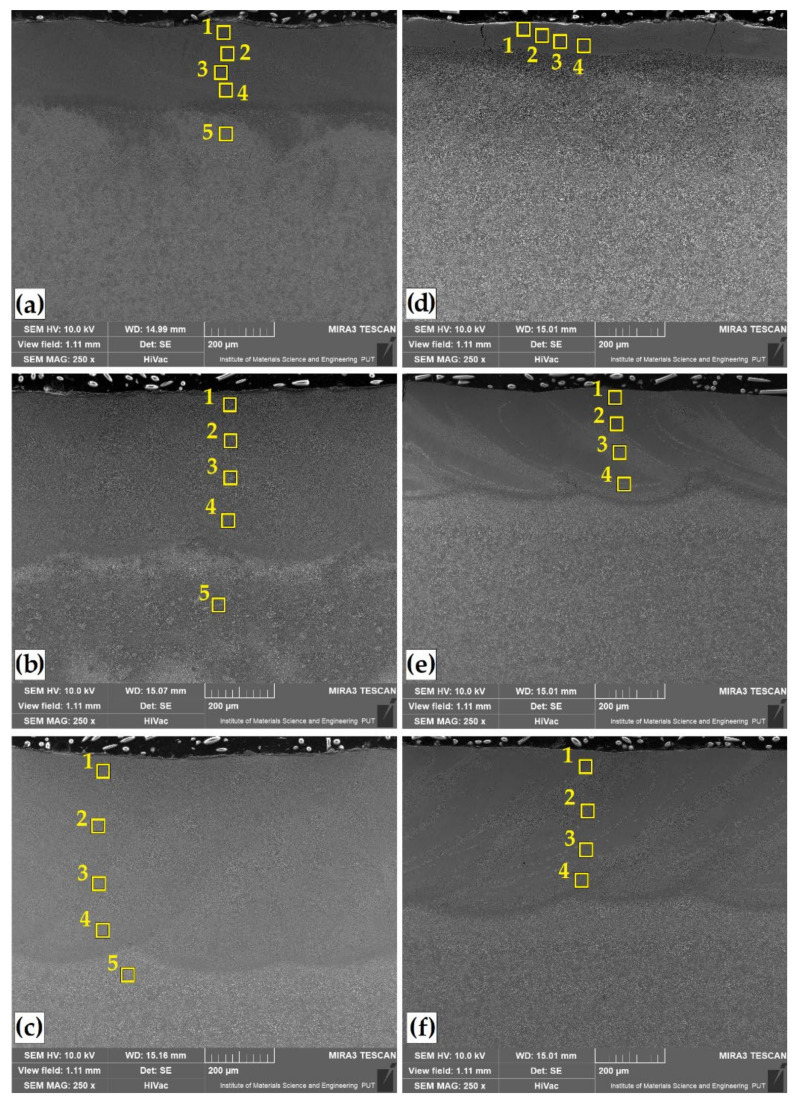
Microstructure of laser tracks: 100%Cr–550 W (**a**); 100%Cr–750 W (**b**); 100%Cr–950 W (**c**); 100%B–550 W (**d**); 100%B–750 W (**e**); 100%B–950 W (**f**). Numbered squares show where the EDS analysis was performed.

**Figure 4:**
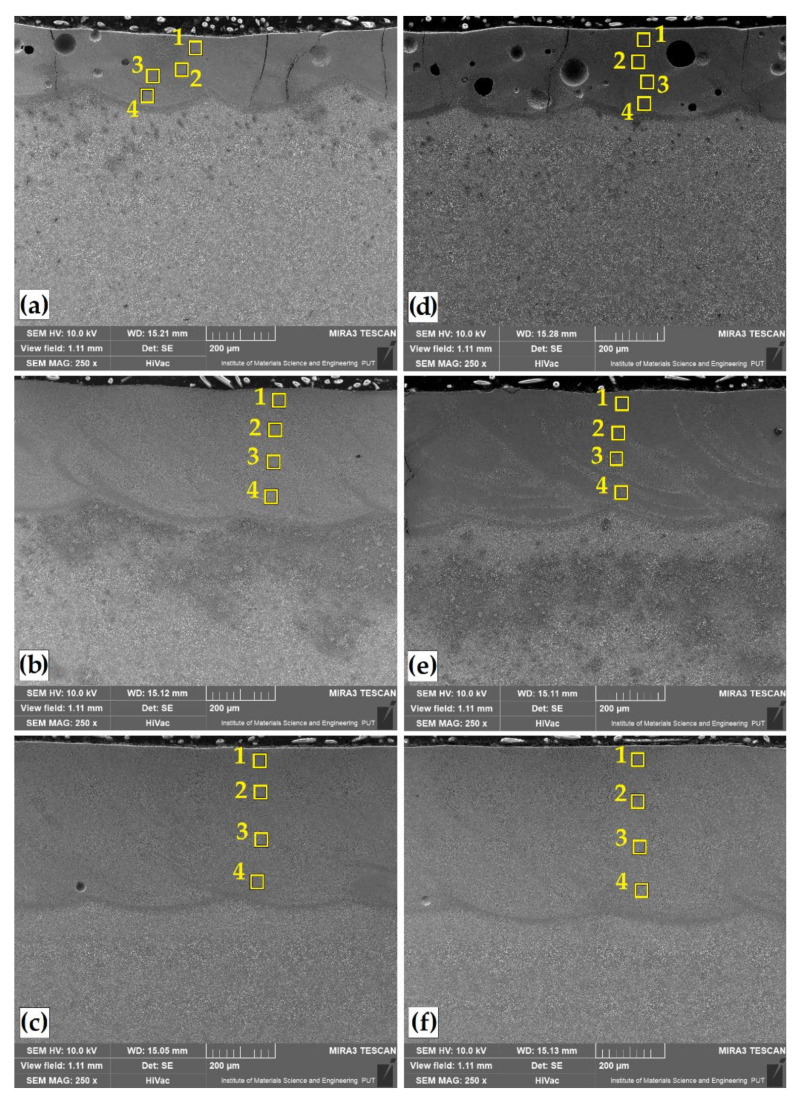
Microstructure of laser tracks: 25%Cr/75%B–550 W (**a**); 25%Cr/75%B–750 W (**b**); 25%Cr/75%B–950 W (**c**); 50%Cr/50%B–550 W (**d**); 50%Cr/50%B–750 W (**e**); 50%Cr/50%B–950 W (**f**). Numbered squares show where the EDS analysis was performed.

The microstructures of Cr, B and Cr-B coatings produced by laser alloying are shown in Figure 3 and Figure 4. Observations of the microstructure were made for coatings obtained at three different laser beam powers. A distinctive change in the thickness of the remelted zone (MZ) was visible. The depth values of this zone are summarized in Table 4. Figure 3a,c shows the microstructure of the layer obtained by laser remelting chromium precoat, while Figure 3d–f shows the microstructure of the coatings produced by laser remelting boron precoat. Figure 4 shows surface layers obtained by remelting the initial coatings produced with different proportions of chromium and boron. The ratio of these two elements was 25%Cr/75%B (Figure 4a–c) or 50%Cr/50%B (Figure 4d–f). As a result of chromium laser alloying, tracks with deepest fusion were obtained, in which no cracks or porosity was found. On the other hand, the use of boron as an element enriching the substrate leads to the obtaining of laser tracks of the smallest depths. Additionally, when using the lowest laser beam power (550 W), cracks were visible in the remelted zone, which propagated along its entire depth. Increasing the laser beam power, and thus obtaining a deeper fusion, contributed to removal of microstructure imperfections. It should be assumed that the obtained results are influenced by initial characteristics of these elements, i.e., thermal conductivity and heat capacity. Chromium, like iron, has similar values of thermal conductivity coefficient, in contrast to boron. Each of these elements is characterized by a different hardenability. In steel, a small addition of boron significantly increases its hardenability; therefore, the occurrence of cracks with its large amount is inevitable.

This study involved a modification of the surface layer with these two elements in order to obtain a coating with the best properties. It was found that increasing the boron content at the expense of chromium results in remelting, which is greater than that of the boron coating itself but lesser than that of the chromium coating. The coatings obtained with the laser beam power of 750 and 950 W were characterized by a uniform microstructure devoid of porosity and cracks. The thickness of these coatings was uniform. There were no significant thickness deviations in the axis of laser tracks and at their interfaces. It can be concluded that only too low laser beam power did not have a positive effect on surface layers obtained as numerous cracks and porosities were visible there. It can be assumed that insufficient laser beam power prevented uniform mixing of boron and chromium components with the substrate. Similar conclusions are presented in [34], where the author observed that the modification of surface layer with boron and another element or compound is advantageous. He stated that what is important is density of elements or compounds introduced simultaneously with boron in laser alloying. Too great a difference in density between boron powder and another powder could result in powder settling at the bottom of the track without any distribution in the remainder of the remelted zone. To avoid this phenomenon, a powder with lower density can be added. Addition of powder that is lighter than iron derived from the substrate can slow down the convection flow with a better distribution into the pool.

In order to analyze the distribution of the introduced chemical elements in the surface layer obtained, the chemical composition was tested using the EDS method. The measurement sites for each of the tested laser tracks containing elements modifying Vanadis 6 steel surface layer are marked with squares in Figure 3 and Figure 4, and the results are presented in Table 5.

In the case of a sample laser alloyed with chromium, the amount of chromium decreases as the power of the laser beam increases. For a power of 550 W, its amount fluctuates in the range of approximately 15 wt.% and decreases to approximately 9 wt.% at 950 W. A similar dependence also existed as a result of remelting the initial boron coating with a laser beam. With the laser beam power of 550 W, the boron content in the entire area of the remelted zone was in the range of approximately 9 wt.%, while the increase in laser beam power resulted in its reduction to approximately 2.5 wt.%

When Cr-B mixtures were used, the amount of elements introduced changed slightly. For chromium, no significant change in the percentage was noticed when a variable laser beam power was used. The percentage of chromium in the 25%Cr/75%B coating was in the range of approximately 9 wt.%, while it was approximately 10 wt.% for the 50%Cr/50%B coating. In the case of boron, it was found that with the increase in laser beam power, its share in the 25%Cr/75%B coating changes from approximately 6 wt.% (550 W) to approximately 3 wt.% (950 W). A similar relationship in the case of boron occurred for the 50%Cr/50%B coating, where its share changed from approximately 4 wt.% (550 W) up to approximately 2 wt.% (950 W).

**Figure 5:**
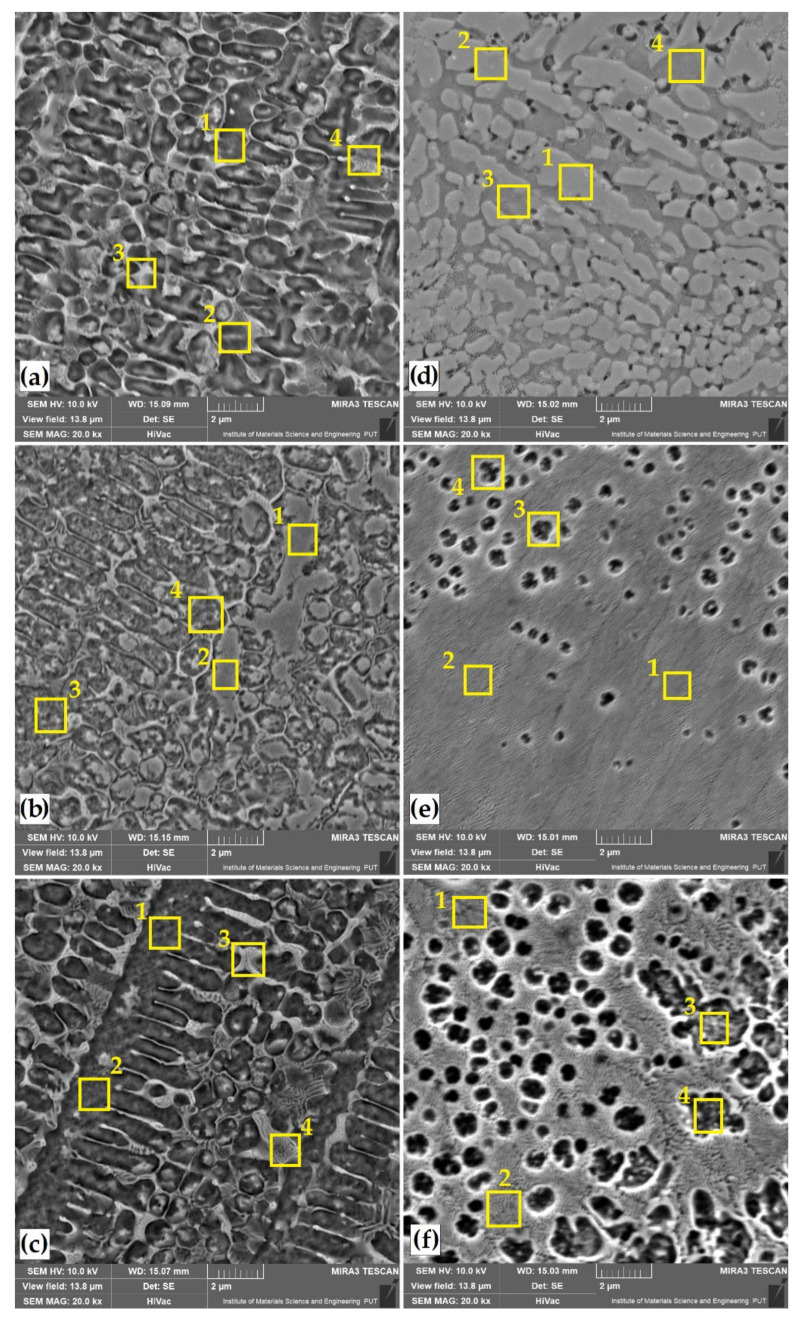
Microstructure of remelted zone of laser tracks: 100%Cr–550 W (**a**); 100%Cr–750 W (**b**); 100%Cr–950 W (**c**); 100%B–550 W (**d**); 100%B–750 W (**e**); 100%B–950 W (**f**). Numbered squares show where the EDS analysis was performed.

**Figure 6:**
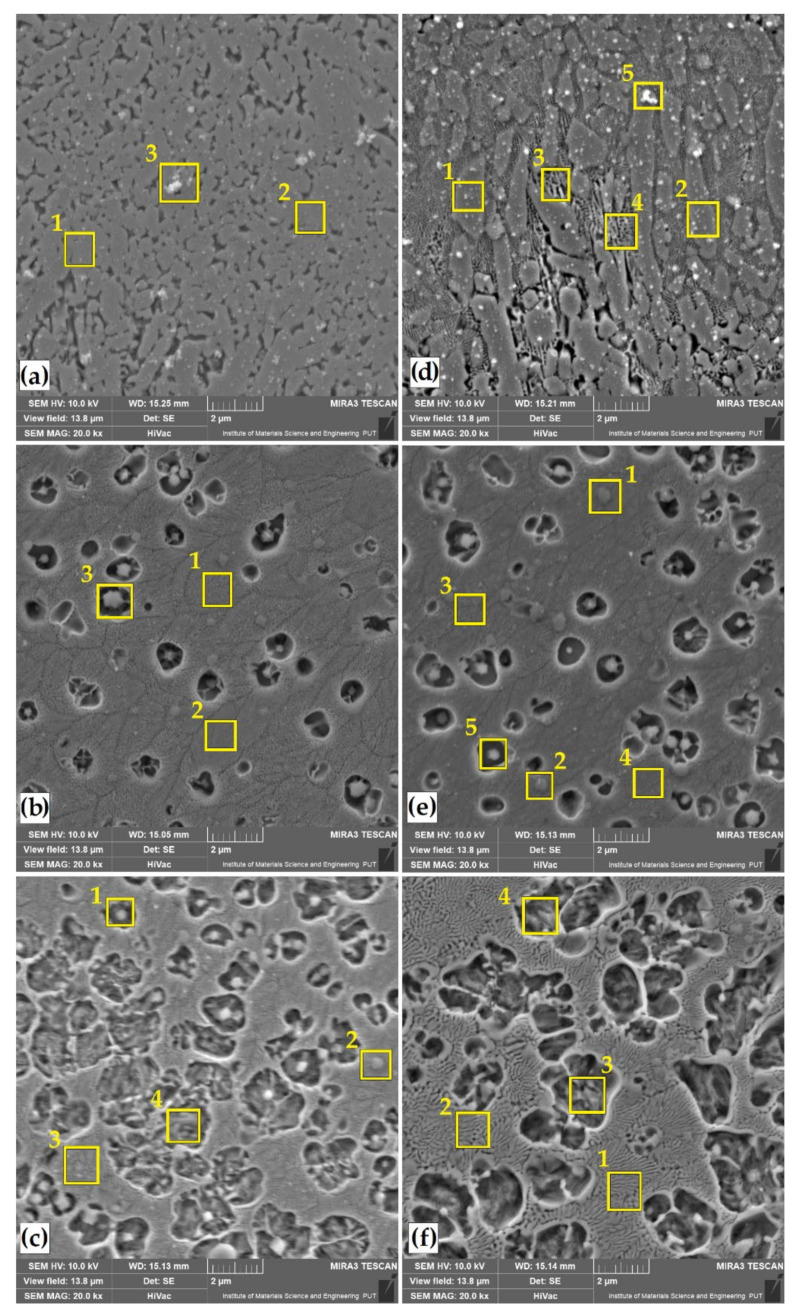
Microstructure of remelted zone of laser tracks: 25%Cr/75%B–550 W (**a**); 25%Cr/75%B–750 W (**b**); 25%Cr/75%B–950 W (**c**); 50%Cr/50%B–550 W (**d**); 50%Cr/50%B–750 W (**e**); 50%Cr/50%B–950 W (**f**). Numbered squares show where the EDS analysis was performed.

Changes were also observed in the percentage of vanadium and carbon. Here, their percentage was influenced by laser beam parameters. The magnitude of laser beam power might have affected the remelting of carbides derived from Vanadis 6 steel substrate. This phenomenon was examined by observing the microstructure of remelted zone at high magnifications (Figure 5 and Figure 6). It can be concluded that the increase in laser beam power increases the amount of iron derived from the substrate in the resulting remelted zone. In the samples analyzed, with the types of coatings and laser parameters applied, no visible heat-affected zone was observed. It is best visible in Figure 4e.

Figure 5 and Figure 6 show enlarged areas from central locations of laser tracks in Figure 3 and Figure 4. The choice of magnification site was justified by the fact that in visual observation of laser tracks the distribution of the introduced elements was uniform.

Some swirls in the chemical composition could be observed in Figure 3e and Figure 4e, but chemical composition tests did not confirm any significant differences. In the case of a chromium laser alloyed coating, the structure obtained is a solid solution of chromium in iron. The microstructure exhibits dendrites, the size of which depends on solidification rate of the coating. First-order dendrites from which second-order dendrite limbs extend are clearly visible. In all laser beam powers applied, structure growth is uniform and correlated to laser beam power magnitude used (Figure 5a–c).

In the case of boron alloyed laser coating, there are significant differences in the microstructure obtained. In all the cases, the microstructure consisted of boron–martensite eutectic, but its share depended on laser beam power applied. For 550 W laser beam power, it can be concluded that we are dealing with a hypereutectic structure (borides + Eu) (Figure 5d). Increasing laser beam power to 750 W resulted in obtaining areas corresponding to both the hypereutectic and eutectic structure (Eu +Feα) (Figure 5e). On the other hand, when using the highest power density, the structure obtained had the character of a boron–martensite eutectic (Figure 5f).

A similar structure after laser boriding with a fine eutectic with very fine primary equiaxial dendrites was observed in [34]. The authors stated that when carbon borides are replaced with boron powder, the cracking phenomenon disappears due to carbon content reduction. It appears that laser remelting of boron coatings gives very fine microstructures uniformly distributed in the entire depth of remelting zone, with a hardness reaching 1200 HV without cracking and without brittle-phase FeB. Mixing chromium and boron with remelted substrate is ensured by convection flows resulting from surface tension (Marangoni effect) and buoyancy effects. The thermal gradient between the region located under the laser beam and the edges of the track creates a surface-tension gradient, which induces the liquid flow. The homogeneity is higher when laser power is high and liquid viscosity low. Additionally, low thermal conductivity can increase thermal gradient and accelerate convection flow. Boron addition, with a low thermal conductivity and a low density, causes good mixing of the pool in chromium powder and iron from steel substrate [34]. In [20], it was shown that introduction of a low amount of boron into the melt, which occurs at a small thickness of the coat or in deep remelting, leads to formation of a structure that consists of primary dendrites (very likely an α-phase) and a boride eutectic (Eu). The structure of the boronized zone is hypoeutectic and has an αFe + Eu form. With the introduction of a high amount of boron into the remelt, the remelted zones have a structure that consists of primary boride crystals and a eutectic. The structure of the remelted zone is hypereutectic (Eu + borides), and the primary borides can be of various shapes, i.e., branched, round, prismatic or angular. In [37], the authors presented the structure evolution upon non-equilibrium solidification of bulk undercooled Fe–B system. In their study, they described a characteristic microstructure (hypereutectic, eutectic and hypoeutectic) during high undercooling and hypercooling using molten B_2_O_3_ glass. Some of the microstructures obtained by laser boriding are similar. However, it should be kept in mind that the processing method and conditions are slightly different.

The use of the Cr-B mixture for surface modification significantly influenced the changes in the microstructure. Here, too, the share of boron–chromium eutectic changes depended on laser beam parameters used. The most dense microstructure is obtained at the lowest laser beam power (Figure 6a,d).

However, this microstructure has numerous imperfections that can be observed in Figure 4a,d. An increase in laser beam power contributes to the achievement of an even microstructure. The use of a 50%Cr/50%B coating shows the best results in terms of microstructure. In [32], the authors presented laser alloying of 45 steel with chromium and boron carbide. The authors stated that when chromium is added to the coating, the microstructure of the fusion zone changes. The boride components (50%B_4_C + 50%Cr) form a ledeburite-like microstructure.

**Figure 7:**
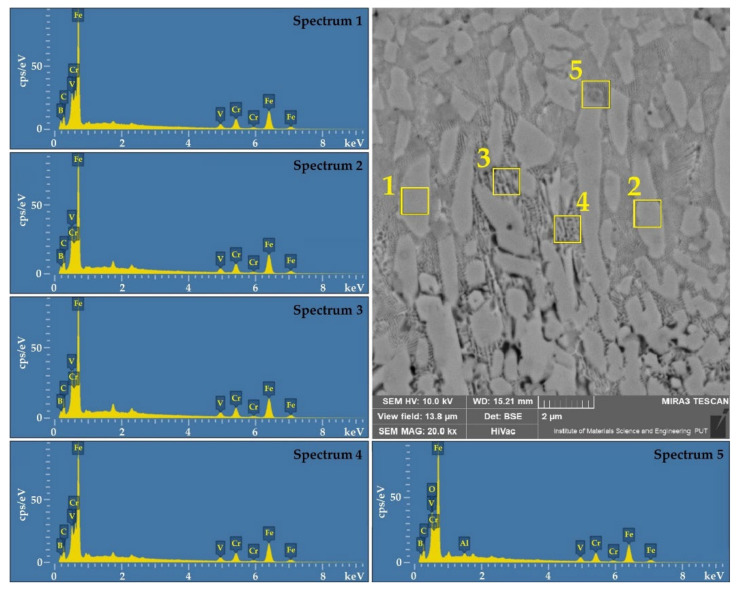
EDS spectra of point analysis of remelted zone of laser track: 50%Cr/50%B–550 W.

The places of the remelted zone for which the EDS point microanalysis tests were conducted are marked with squares in Figure 5 and Figure 6, and the results are presented in Table 6. Additionally in Figure 7, the EDS spectra of point analysis in the remelted zone are shown for one of the coatings (50%Cr/50%B–550 W).

As for chromium coating, an increased chromium content was found in the areas of dendrites, the amount of which ranged from 20 wt.% (550 W) to approximately 10 wt.% (950 W). Such content corresponded to the intermetallic phases of the (Cr_1-x_Fe_x_)_7_C_3_, (Fe_1-x_Cr_x_)_3_C, (Ce_1-x_Fe_x_)_23_C_6_ type. In [23], the author also showed that the area of the remelted zone is chromium-enriched. Chromium segregation proceeds to cell boundaries, where an increased chromium content was found. In the boron coating at a lower laser beam power, approximately 9 wt.% boron content was observed in dendritic areas, which corresponds to the content of iron boride Fe_2_B according to the Fe-B diagram. Increasing laser beam power reduces boron content. The phases that can be attributed to this content are Fe_3_(C,B) or Fe_23_(C,B)_6_. As a result of the application of the Cr-B mixture in the remelting zone, a chemical composition was obtained corresponding to the complex phase (Cr,Fe)_2_B, (Fe,Cr)_3_(C,B) [38,39,40,41,42,43,44]. In the photos of microstructures 6a and 6d, white precipitates are visible. The chemical composition tests using the EDS method confirmed that they are Al_2_O_3_ coming from sample polishing. They might have remained on the cross-sectional surface of the sample due to porosity and cracks, which made it impossible to rinse it thoroughly.

**Figure 8:**
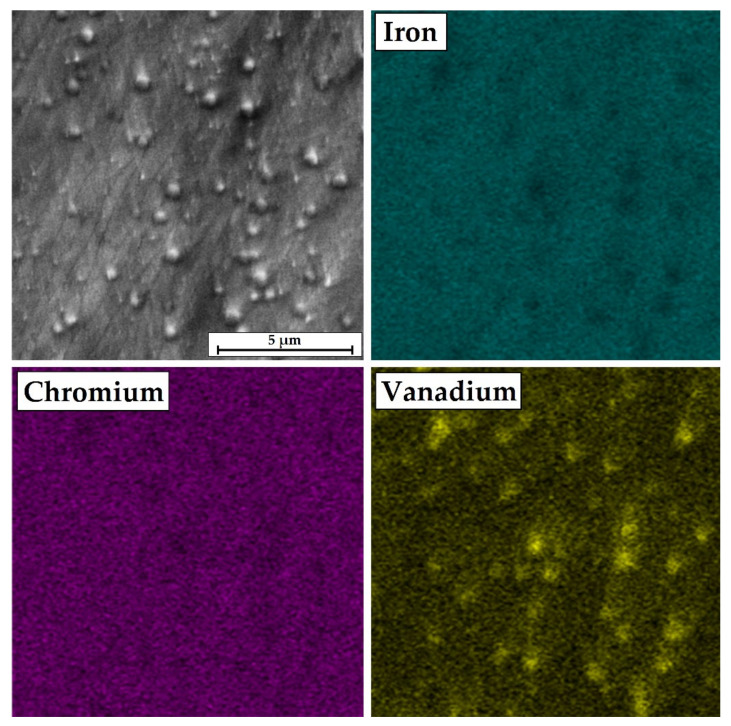
EDS map of microstructure of remelted zone of laser tracks 50%Cr/50%B–750 W.

Figure 8 shows the EDS mapping of the chemical composition for the 50%Cr/50%B 750 W fused coating zone. Unmelted vanadium carbides derived from Vanadis 6 steel substrate are visible here. The share of vanadium and carbon depends on the parameters that affect heat accumulation and thus affect the remelting or non-remelting of vanadium carbides. In Vanadis 6 steel, apart from vanadium, chromium carbides are also present, but they were not observed in the remelted zone, which may indicate their dissolution. It is influenced by the melting point of individual carbides, where for VC it is 2810 °C, and for Cr_3_C_2_ it is over 900 °C lower.

### 3.2. Microhardness Profiles

**Figure 9:**
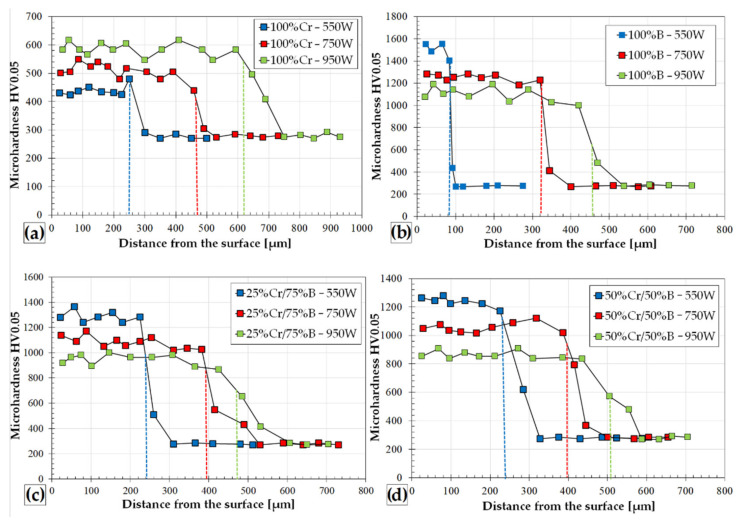
Microhardness profiles of laser tracks with different precoat: 100%Cr–550 W, 750 W, 950 W (**a**); 100%B–550 W, 750 W, 950 W (**b**); 25%Cr/75%B–550 W, 750W, 950W (**c**); 50%Cr/50%B–550 W, 750 W, 950 W (**d**).

Figure 9 shows microhardness profiles of laser alloyed Cr, B and Cr-B coatings. In the case of a chromium laser alloyed coating, the microhardness is in the range of approximately 400–600 HV0.05 (Figure 9a). Low laser beam power resulted in microhardness of 400 HV0.05, which was lower than the measurement in the area of the heat-affected zone. The increase in the power of the laser beam in which a significant amount of chromium was mixed with the base material (iron) increased the hardness to approximately 600 HV0.05. In each of the analyzed cases, the microhardness along the track cross-section was uniform. The diagram in Figure 9b shows the microhardness profiles for the laser borided layer. The highest hardness, up to approximately 1600 HV0.05, was obtained for the thinnest coating. For the lowest power of the laser beam, a large microhardness gradient between the layer and the substrate was visible. The increase in laser beam power resulted in obtaining slightly lower microhardness values of the order of 1200–1100 HV0.05, which fluctuated in this range across the entire depth of the remelted zone. However, the hardness gradient between the layer and the substrate was quite large. Figure 9d shows the microhardness profiles for the 50%Cr/50%B coating. Despite the satisfactory microhardness results, the coating produced with the laser beam power equal to 550 W should be rejected due to its defective microstructure (Figure 4d).

The mixture of Cr and B at a higher laser beam power gives satisfactory results because the hardness is uniform across the cross-section of the layer. For the 750 W laser beam power, it is approximately 1000 HV0.05, and for 950W, it is approximately 900 HV0.05. The increase in the proportion of boron at the expense of chromium in the 25%Cr/75%B coating (Figure 9c) contributed to higher hardness values of 1300 HV0.05 (550 W), 1100 HV0.05 (750 W) and 1000 HV0.05 (950 W); however, within the track, hardness values fluctuate in the range of 1000 HV0.05. The author of [20] stated that the obtained microhardness depends on the obtained microstructure, which is influenced by the amount of boron introduced in area of remelted zone and the steel grade. The microhardness of the layer may vary from approximately 500 to 1200 HV. In [21], the boronized layers of 60% FeB–40% B_2_O_3_ composite powder were characterized by a high hardness values from 1134 to 1220 HV. Morimoto et al. stated that the hardness of the boronized layer is dependent on laser power. The boronized layers formed at laser power of 275 W and scanning speed of 0.6 mm/s had a low hardness of 854 HV. This is due to increase in Fe from the substrate mixed with the boron in the layer formed. The best microhardness obtained gradually decreases from 1500 to 850 HV from the surface and becomes 310 HV in the substrate. In [32], the authors stated that as the amount of chromium contained in the coating increases, the amount of borides diminishes and, similarly, hardness decreases.

### 3.3. Corrosion Resistance

**Figure 10:**
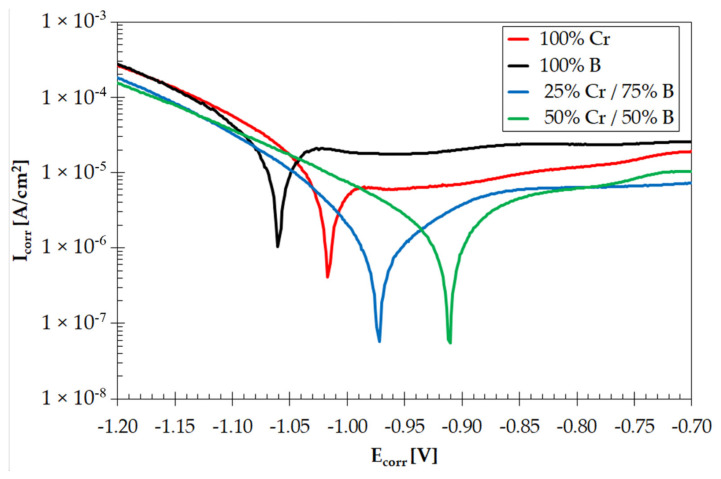
Corrosion resistance tests results of coatings produced using laser alloying methods (750 W).

Figure 10 shows the Tafel curves after the corrosion resistance tests using the potentiodynamic method. The 50%Cr/50%B coating shows the best corrosion resistance, and the worst corrosion resistance was demonstrated by boron coating, which has a higher corrosion potential and higher corrosion current values. The concept of adding chromium to the boron precoat seems promising. Chromium, as a chemical element increasing the corrosion resistance of steel, has also improved corrosion resistance of coatings obtained. Additionally, it can be stated that in Cr-B coatings, this resistance increases with increasing chromium content. Detailed results of corrosion potential and corrosion rate are summarized in Table 7.

B-Cr coatings produced using powder mixture of chromium and boron were characterized by good corrosion resistance in comparison to chromium or boron coatings. It is assumed that when the corrosion curves are shifted towards greater potential and lower corrosion current, the corrosion resistance of tested material increases. In described cases, these conditions are met by a graph plotted for a coating produced using 50%Cr and 50%B.

**Figure 11:**
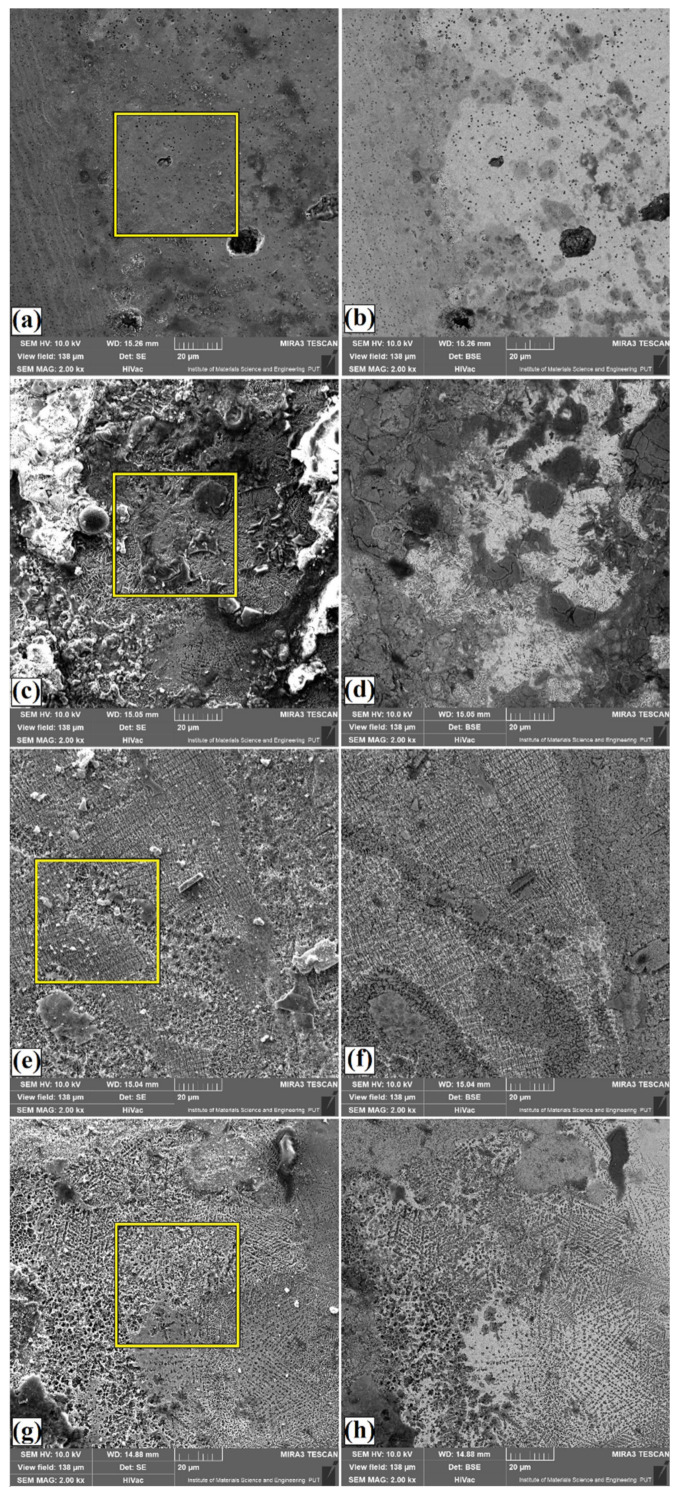
Surface condition after corrosion tests of laser tracks with different precoat for laser power 750 W: 100%Cr (**a**); 100%Cr–enlarged the area marked with a square (**b**); 100%B (**c**); 100%B–enlarged the area marked with a square (**d**); 25%Cr/75%B (**e**); 25%Cr/75%B–enlarged the area marked with a square (**f**); 50%Cr/50%B (**g**); 50%Cr/50%B–enlarged the area marked with a square (**h**).

**Figure 12:**
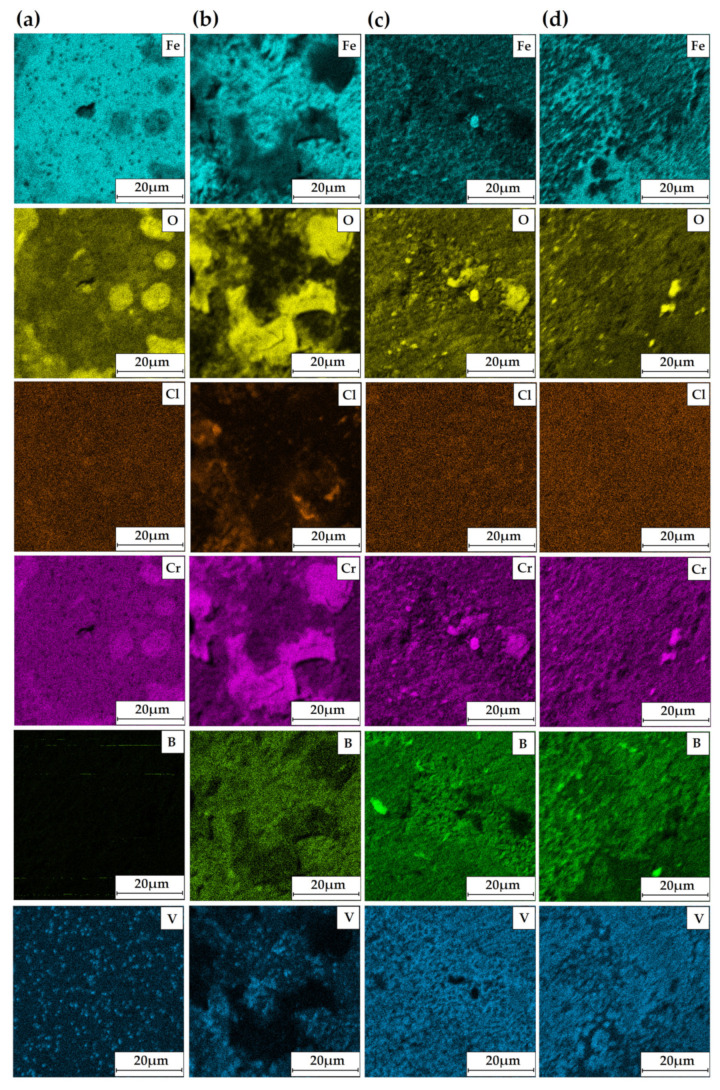
EDS mapping of surface condition after corrosion tests of laser tracks with different precoat for laser power 750 W: 100%Cr (**a**); 100%B (**b**); 25%Cr/75%B (**c**); 50%Cr/50%B (**d**).

Surface conditions after corrosion resistance tests for coatings produced by applying different types of precoats and laser beam power 750 W are shown in Figure 11a–d (SE and BSE contrast). The EDS mappings of surfaces after corrosion tests are shown in Figure 12a–d. The areas marked with squares in Figure 11 were examined. The coating produced using precoat of chromium was characterized by a small number of corrosion pits, which were characterized by a quite large size (Figure 11a). Increased oxygen content can be observed mainly in the boron coating (Figure 11b and Figure 12b). Therefore, it can be concluded that this coating was more susceptible to corrosion. On the coating containing chromium and boron, corrosion pits were observed, but they were evenly distributed. Therefore, it can be concluded that the production of Cr-B coating increased corrosion resistance compared to the chromium or boron coatings (Figure 11c,d and Figure 12c,d). The basic chemical elements from the coatings, oxygen responsible for the formation of oxides and chlorine from the corrosive solution used for the tests were taken into account in the EDS mappings. Figure 12d shows the coating produced using the precoat consisting of 50%Cr and 50%B. The oxide products appeared on the entire observed area, and the distribution of oxygen was relatively even. It can be concluded that an oxide layer was created on the specimen surface.

**Figure 13:**
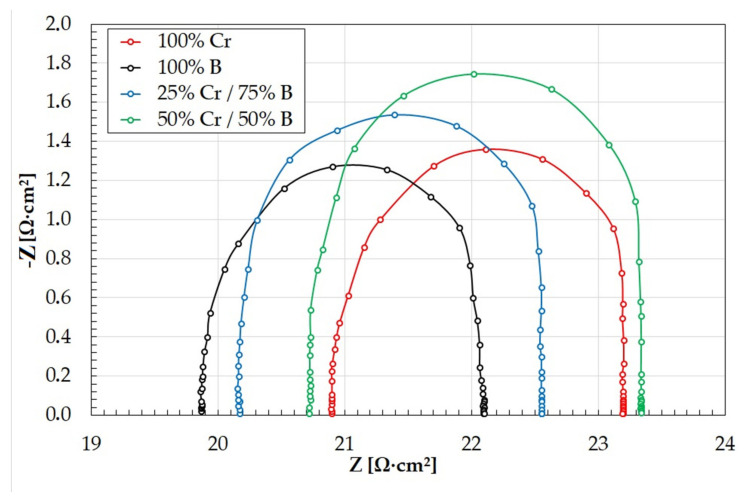
Electrochemical impedance spectroscopy diagrams of produced coatings (Nyquist plots).

**Figure 14:**
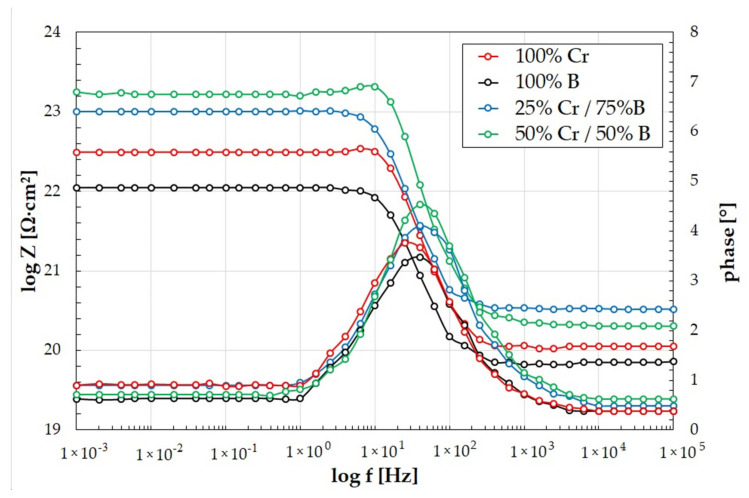
Electrochemical impedance spectroscopy diagrams of produced coatings (Bode plots).

Electrochemical impedance spectroscopy diagrams are presented in Figure 13 and Figure 14. The amplitude–phase characteristic in the form of Nyquist plots is semicircular (Figure 13). The larger diameter of the semicircle indicates a higher electric current resistance at the interface between the coating and solution. In the analyzed coatings, the highest corrosion resistance was observed for 50%Cr/50%B coating. The smallest diameter of the semicircle indicates the lowest corrosion resistance (100%B coating). This phenomenon is confirmed by the anodic polarization results. The EIS results are closely correlated with the data obtained for the potentiodynamic corrosion test. The impedance results in the form of a Bode plot allow tracking changes of the impedance in a wide frequency range. The obtained Bode plots have similar shapes for all coatings tested, and they are symmetrical and have one maximum located in the low frequency range (Figure 14). The highest value of the impedance modulus was obtained for 50%Cr/50%B coating (Figure 14). This means that a layer of corrosion products formed on the specimen effectively protects the coating against further degradation, which is confirmed by the EDS results (Figure 12d). The lowest value of the impedance modulus was obtained for 100%B coating, which is also confirmed by study the chemical composition of the specimen surface after corrosion tests.

## 4. Conclusions

This study analyzed changes in microstructure, chemical composition, microhardness, and corrosion resistance of Cr-B coatings produced on Vanadis 6 tool steel with the use of a diode laser beam. Based on the analyses, the following conclusions were drawn:

1. It was found that laser processing of a precoat containing boron and chromium results in a greater remelting zone than laser processing of a precoat consisting of boron or chromium separately. Proper selection of laser beam parameters is very important. The coatings obtained with the laser beam power of 750 and 950 W were characterized by a uniform microstructure devoid of porosity and cracks, but too low laser beam power makes it impossible to mix the boron and chromium components evenly.
2. The microstructure of laser alloyed chromium consists of a solid solution of chromium in iron. The microstructure of laser alloyed boron coating consists of boron–martensite eutectics, but their share depends on the laser beam power used. The use of the Cr-B mixture contributes to the formation of boron–chromium eutectic, the amount of which depends on the laser parameters used.
3. The addition of chromium to the boron precoat increases corrosion resistance, and the increase in chromium content improves this property.

## Figures and Tables

**Table 1:** Chemical composition of Vanadis 6 tool steel used (wt.%).

C	Si	Mn	Cr	Mo	V	Fe
2.1	0.9	0.4	6.7	1.5	5.4	Balance

**Table 2:** Composition of precoat in the form of paste.

Precoat Composition	Cr 100%	B 100%	Cr and B Mixture 50%/50%	Cr and B Mixture 25%/75%
Chemical element (g)	2.0	1.0	1.0/1.0	1.0/3.0
Sodium water glass (mL)	0.5	0.5	0.5	1.0
Distilled water (mL)	1.0	1.5	1.5	3.0

**Table 3:** Parameters of laser alloying.

Laser Beam Power (W)	Scanning Speed (mm/s)	Exposure Time (s)	Fluence (J/mm^2^)	Overlapping (%)
550	50	0.02	14	60
750			19	
950			24	

**Table 4:** Average dimensions of remelted zone of laser tracks.

P (W)	Type of Coating			
	100% Cr	100% B	25%Cr/75%B	50%Cr/50%B
	Depth of MZ along the Laser Track Axis (µm)			
550	235	85	230	247
750	470	324	390	405
950	617	453	470	512

**Table 5:** Results of EDS from areas analysis of produced coatings marked in Figure 3 and Figure 4 (wt.%).

Sign	No.	Fe	Cr	B	V	C
100%Cr 550 W	1	55.4	17.9	-	13.9	12.9
	2	58.5	15.6	-	13.5	12.4
	3	58.5	15.4	-	13.2	12.9
	4	63.8	12.9	-	11.3	12.0
	5	83.5	8.9	-	2.5	5.2
100%Cr 750 W	1	66.2	11.7	-	12.3	9.8
	2	73.1	8.6	-	8.8	9.4
	3	71.4	11.0	-	9.5	8.1
	4	66.5	11.3	-	10.4	11.7
	5	73.0	9.5	-	8.8	8.8
100%Cr 950 W	1	74.3	9.0	-	8.5	8.1
	2	71.9	9.9	-	9.4	8.8
	3	72.8	9.9	-	8.7	8.6
	4	74.5	9.4	-	7.9	8.2
	5	77.2	8.6	-	8.1	6.1
100%B 550 W	1	73.2	6.5	9.3	4.2	6.7
	2	75.8	6.0	9.0	4.7	4.5
	3	73.9	7.4	7.1	6.3	5.2
	4	71.9	8.8	6.4	7.3	5.6
100%B 750 W	1	75.2	7.9	3.2	7.9	5.9
	2	71.6	8.0	3.6	6.8	9.9
	3	72.1	8.4	4.2	6.7	8.5
	4	74.0	7.7	3.8	6.7	7.8
100%B 950 W	1	73.8	8.5	3.2	8.2	6.2
	2	73.3	8.4	2.5	8.7	7.2
	3	69.5	9.1	2.3	10.7	8.5
	4	73.5	8.3	2.1	8.7	7.4
25%Cr/75%B 550 W	1	73.9	9.5	6.4	5.3	4.9
	2	76.1	8.9	7.6	4.0	3.4
	3	77.3	8.8	5.9	4.1	3.9
	4	73.0	10.1	3.8	6.4	6.7
25%Cr/75%B 750 W	1	70.6	9.6	4.3	7.4	8.1
	2	73.7	9.3	3.5	6.6	6.9
	3	73.7	8.6	3.6	6.9	7.2
	4	75.8	8.6	3.6	5.0	6.9
25%Cr/75%B 950 W	1	72.3	9.3	3.6	8.2	6.6
	2	66.2	9.5	2.7	11.4	10.2
	3	71.9	8.6	3.0	8.8	7.7
	4	71.1	9.5	3.5	8.6	7.3
50%Cr/50%B 550 W	1	69.2	10.8	4.2	9.2	6.6
	2	69.4	11.2	3.9	8.5	7.0
	3	72.1	10.1	4.1	7.2	6.5
	4	69.3	10.8	4.1	8.3	7.5
50%Cr/50%B 750 W	1	69.6	11.0	2.7	9.3	7.4
	2	71.6	10.1	3.1	8.5	6.6
	3	72.6	10.1	3.5	6.7	7.0
	4	71.3	10.4	3.5	8.1	6.7
50%Cr/50%B 950 W	1	68.8	10.3	2.7	10.4	7.7
	2	72.7	10.0	2.9	8.2	6.2
	3	69.9	9.0	2.2	10.2	8.7
	4	71.8	8.4	1.7	9.2	9.0

**Table 6:** Results of EDS point analysis of produced coatings marked in Figure 5 and Figure 6 (wt.%).

Sign	No.	Fe	Cr	B	V	C	O	Al
100%Cr 550 W	1	69.7	11.0	-	8.2	11.0	-	-
	2	68.1	12.6	-	9.3	10.0	-	-
	3	49.3	20.6	-	14.0	16.1	-	-
	4	50.7	18.5	-	17.0	13.8	-	-
100%Cr 750 W	1	68.1	12.1	-	8.5	11.3	-	-
	2	67.1	14.3	-	8.2	10.3	-	-
	3	77.4	8.0	-	5.1	9.5	-	-
	4	77.9	8.1	-	4.8	9.1	-	-
100%Cr 950 W	1	78.2	4.1	-	7.9	9.8	-	-
	2	77.5	4.7	-	8.2	9.6	-	-
	3	72.2	11.3	-	6.2	10.3	-	-
	4	73.9	9.5	-	6.8	9.8	-	-
100%B 550 W	1	75.9	7.6	8.9	3.7	3.8	-	-
	2	75.4	7.4	8.7	4.3	4.2	-	-
	3	73.1	7.8	5.8	5.4	8.0	-	-
	4	75.1	7.7	4.4	4.0	8.8	-	-
100%B 750 W	1	73.7	8.2	4.5	7.2	6.5	-	-
	2	73.9	8.3	4.4	6.9	6.5	-	-
	3	76.6	7.0	2.9	8.0	5.5	-	-
	4	78.5	7.7	2.5	6.5	4.9	-	-
100%B 950 W	1	72.9	8.8	3.8	7.7	6.9	-	-
	2	72.9	9.4	3.3	7.3	7.1	-	-
	3	76.7	6.5	1.7	9.2	5.8	-	-
	4	78.3	6.2	1.9	5.7	7.9	-	-
25%Cr/75%B 550 W	1	76.3	9.3	6.2	3.5	4.6	-	-
	2	76.2	9.0	7.0	4.2	3.6	-	-
	3	68.1	8.5	5.2	5.9	5.2	5.2	1.5
25%Cr/75%B 750 W	1	73.4	9.0	3.7	6.7	7.2	-	-
	2	75.0	9.0	3.7	5.9	6.5	-	-
	3	54.1	8.9	3.0	23.2	10.7	-	-
	4	56.1	9.1	2.8	22.1	10.0	-	-
25%Cr/75%B 950 W	1	62.3	8.8	3.1	14.3	11.5	-	-
	2	58.0	9.2	3.2	16.8	12.8	-	-
	3	73.9	8.9	4.3	6.1	6.8	-	-
	4	75.4	9.1	3.5	5.8	6.2	-	-
50%Cr/50%B 550 W	1	69.8	13.8	8.0	4.3	4.1	-	-
	2	71.5	12.6	7.4	4.3	4.1	-	-
	3	72.3	12.4	5.4	4.6	5.3	-	-
	4	73.3	12.3	5.5	4.3	4.5	-	-
	5	65.6	12.3	7.2	4.7	5.3	4.4	0.5
50%Cr/50%B 750 W	1	50.8	11.9	2.8	23.5	11.0	-	-
	2	59.6	8.9	3.7	17.8	9.9	-	-
	3	75.6	8.4	3.8	5.6	6.6	-	-
	4	73.4	8.6	3.7	6.8	7.4	-	-
	5	63.2	9.7	2.5	16.0	8.6	-	-
50%Cr/50%B 950 W	1	73.6	11.6	3.7	5.3	5.8	-	-
	2	72.8	11.7	3.6	6.1	5.9	-	-
	3	76.4	8.0	2.2	5.4	8.1	-	-
	4	81.0	7.2	1.3	4.2	6.3	-	-

**Table 7:** Corrosion current and corrosion potential of Cr, B and Cr-B coatings produced using laser alloying of precoat.

Coating Type	Current I_corr_ (A·cm^2^)	Potential E_corr_ (V)
100% Cr	2.77 × 10^−6^	−1.02 × 10^+0^
100% B	7.00 × 10^−6^	−1.06 × 10^+0^
25%Cr/75%B	5.13 × 10^−7^	−9.72 × 10^−1^
50%Cr/50%B	7.42 × 10^−7^	−9.10 × 10^−1^

## Data Availability

Data available on request.
